# The emerging role of anoctamin-1 in cardiac and cerebrovascular diseases

**DOI:** 10.3389/fcvm.2025.1691331

**Published:** 2025-10-24

**Authors:** Jia-qi Zhang, Zeng-jin Wen, Xiao-hua Han, Zhen-kang Qiu, Qing-ya Yang, Yue Qiu

**Affiliations:** ^1^ Interventional Medical Center, The Affiliated Hospital of Qingdao University, Qingdao, Shandong, China; ^2^ Department of Geriatrics, Peking Union Medical College Hospital, Chinese Academy of Medical Sciences and Peking Union Medical College, Beijing, China; ^3^ Department of Physiology, School of Basic Medicine, Qingdao University, Qingdao, China; ^4^ Department of Urology, Qilu Hospital (Qingdao), Cheeloo College of Medicine, Shandong University, Qingdao, China

**Keywords:** hypertension, ANO1, stroke, vascular remodelling, endothelial dysfunction

## Abstract

Cardiocerebral vascular disease has long been the leading cause of morbidity and mortality worldwide. Although there are many effective avenues for preventing and treating cardiocerebral vascular disease, further research is still needed to identify more novel molecular targets for therapeutic intervention. Anoctamin-1 (ANO1), also known as transmembrane protein 16A (TMEM16A), is the molecular identity of calcium-activated chloride channels (CaCCs) and is widely distributed in myocardial cells and the vasculature, including but not limited to the thoracic aorta, mesenteric artery, cerebral artery, and portal vein. ANO1 has many functions in the cardiocerebral vascular system, including cardiac excitability, vascular smooth muscle contraction, and epithelial cell secretion. Aberrant expression or dysfunction of ANO1 is associated with several cardiocerebral vascular diseases, including myocardial ischaemia/reperfusion injury (MIRI), arrhythmias, cardiac fibrosis, hypertension, and stroke. Therefore, this review provides an overview of ANO1, including its structure, distribution, and activation mechanism, and highlights the current knowledge of ANO1 in the pathophysiological process of heart diseases, hypertension, and stroke. We also summarise the pharmacological regulatory target of ANO1, providing promising insights for applying ANO1 inhibitors as cardiac and cerebrovascular therapeutic agents.

## Graphical Abstract

**Figure:**
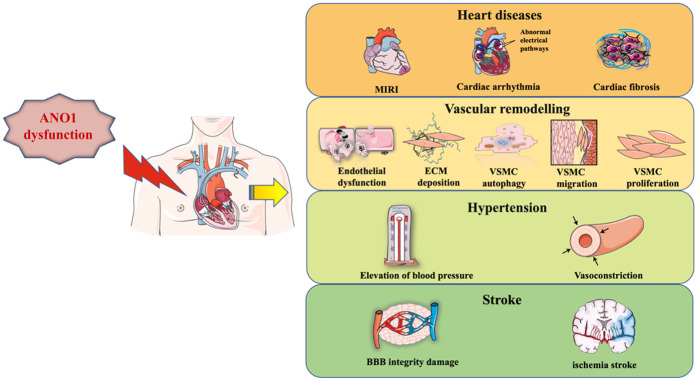

## 1 Introduction

**Figure 1:**
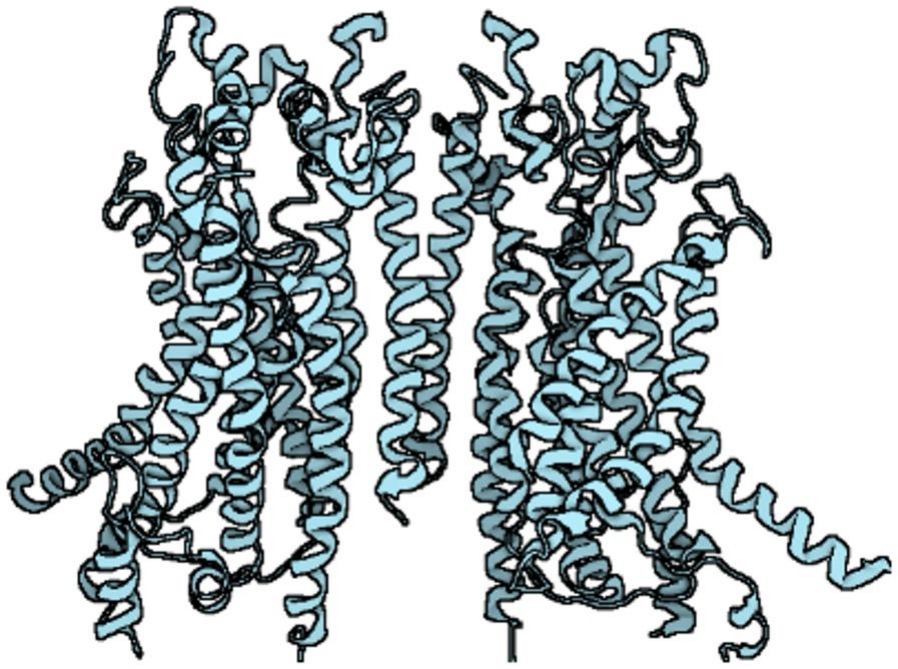
The structure of ANO1.

The anoctamin (ANO)/transmembrane protein 16 (TMEM16) family of membrane proteins consists of ten members, ranging from ANO1 (TMEM16A) to ANO10 (TMEM16K), which play key roles in diverse biological processes, including ion transport, phospholipid scrambling and regulation of other membrane proteins (1, 2). ANO1 was first identified as the gene encoding calcium-activated chloride channels (CaCC) (3–5). Since then, numerous experiments have shown that ANO1 is a homodimer with a quaternary structure comprising two pores (6, 7) and that each subunit consists of cytoplasmic N- and C-terminal domains, ten transmembrane *α*-helix, and extracellular components (8). In addition, a pore is formed in the homodimeric α3-α7 helices, which contain two conserved calcium (Ca^2+^) binding sites, and the inner half of the *α*6 helix acts as a gating element (2, 6). Specifically, certain acidic residues (E654, E702, E705, E734, D738, N650, N651 and N730) are critical for coordination with Ca^2+^ (7–9). The binding of Ca^2+^ to a site located within the transmembrane domain triggers an allosteric process of an *α*-helix, altering the pore conduction and leading to the conduction of an outwardly rectifying current, mainly the efflux of chloride ions (Cl^−^) (as shown in Figure 1) (8).

ANO1, the significant component of the CaCCs in the cardiovascular system, is abundantly distributed in various vascular endothelial cells (ECs) and smooth muscle cells (SMCs) (10). Previous research has shown that activation of ANO1 is gated by cytosolic Ca^2+^ as well as calmodulin, membrane voltage, and external permeant anions (11, 12). Upon exposure to vasoconstrictors (e.g., endothelin-1, angiotensin II), G_q_-protein coupled receptors (G_q_PCRs) are activated, and phosphatidylinositol 4,5-bisphosphate (PIP_2_) is rapidly converted to inositol trisphosphate (IP_3_) and diacylglycerol (DAG). It is generally accepted that ANO1 is directly triggered by Ca^2+^ release from the sarcoplasmic reticulum (SR) via the IP_3_ receptors (13), leading to Cl^−^ efflux and depolarisation of the membrane potential. Voltage-gated L-type calcium channels are then activated, and large amounts of Ca^2+^ enter the cell, causing smooth muscle to contract (14).

**Figure 2:**
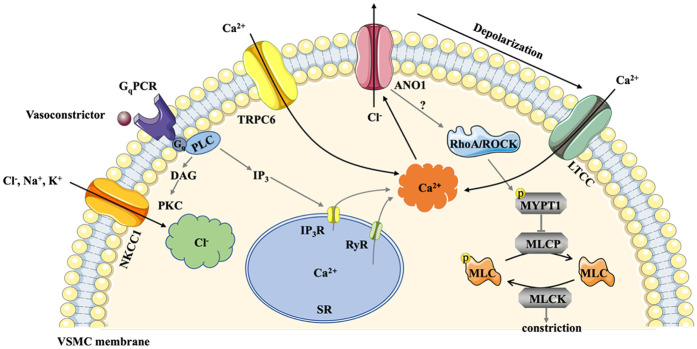
Schematic mechanism of ANO1 activation in VSMC. NKCC1 accumulates substantial Cl^−^ in VSMCs and elevates the driving force for Cl^−^ efflux. Exposed to vasoconstrictors, G_q_PCR is activated, and PIP_2_ is quickly converted to IP_3_ and DAG. ANO1 is directly activated by Ca^2+^ releasing from the SR via the IP_3_R or RyR. TRPC6 channel in cerebral arteries also contributes to elevated Ca^2+^ levels. Activation of ANO1 triggers Cl^−^ outflow and depolarizes the membrane potential, leading to LTCC opening, which causes higher levels of intracellular Ca^2+^. The Ca^2+^- calmodulin complex induces phosphorylation of MLCK to constrict smooth muscle. Additionally, ANO1 is involved in Ang II-induced cerebral constriction via activating RhoA/ROCK signaling pathway, elevating the phosphorylation of MYPT1 and subsequent inhibition of MLCP. ANO1, Anoctamin-1; DAG, diacylglycerol; G_q_PCR, G_q_ protein coupled receptors; IP_3_, inositol trisphosphate; IP_3_R, IP_3_ receptors; LTCC, L-type calcium channels; MLCK, myosin light chain protein kinase; MLCP, myosin light chain phosphorylase; MYPT1:myosin phosphatase targeting subunit 1; NKCC1, Na^+^-K^+^-2Cl^−^ cotransporter 1; PIP_2_, phosphatidylinositol 4,5-bisphosphate; RyR, ryanodine receptors; SR, sarcoplasmic reticulum; VSMC, vascular smooth muscle cells.

**Figure 3:**
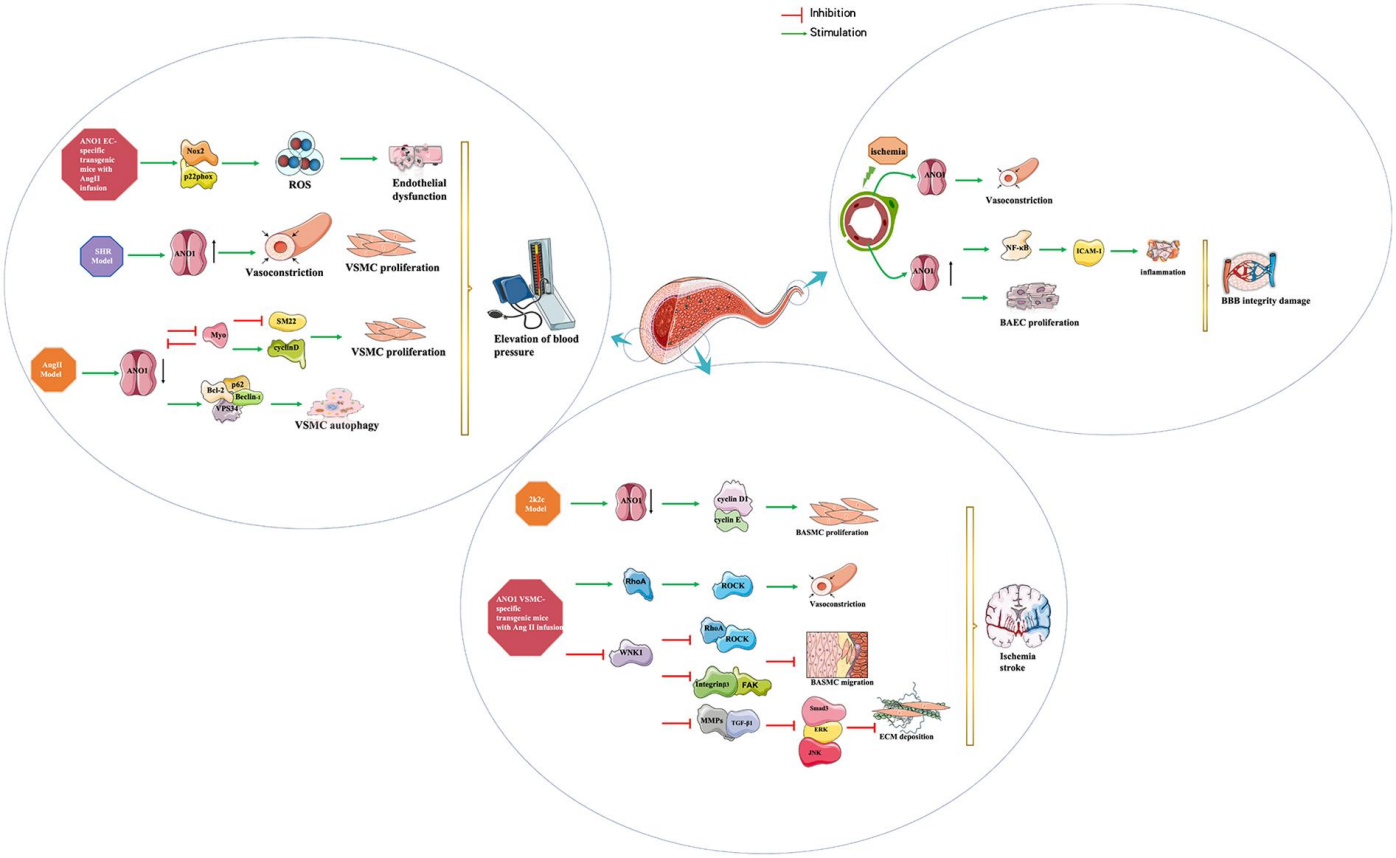
Diagram summarizing the findings on the role of ANO1 in systemic hypertension and stroke. ANO1 plays an important role in the pathophysiology of systemic hypertension, not only in vascular contraction but also in vascular remodelling, including changes in endothelial and VSMC function (left panel). ANO1 also has a pathological effect in cerebrovascular remodelling during the development of hypertension and blood brain barrier injury after stroke. (middle panel and right panel) ANO1, Anoctamin-1;BBB:blood brain barrier; BCEC, brain capillary endothelial cell; BASMC, basilar artery smooth muscle cell; ROS, reactive oxygen species; SHR, spontaneously hypertensive rat; VSMC, vascular smooth muscle cell.

**Figure 4:**
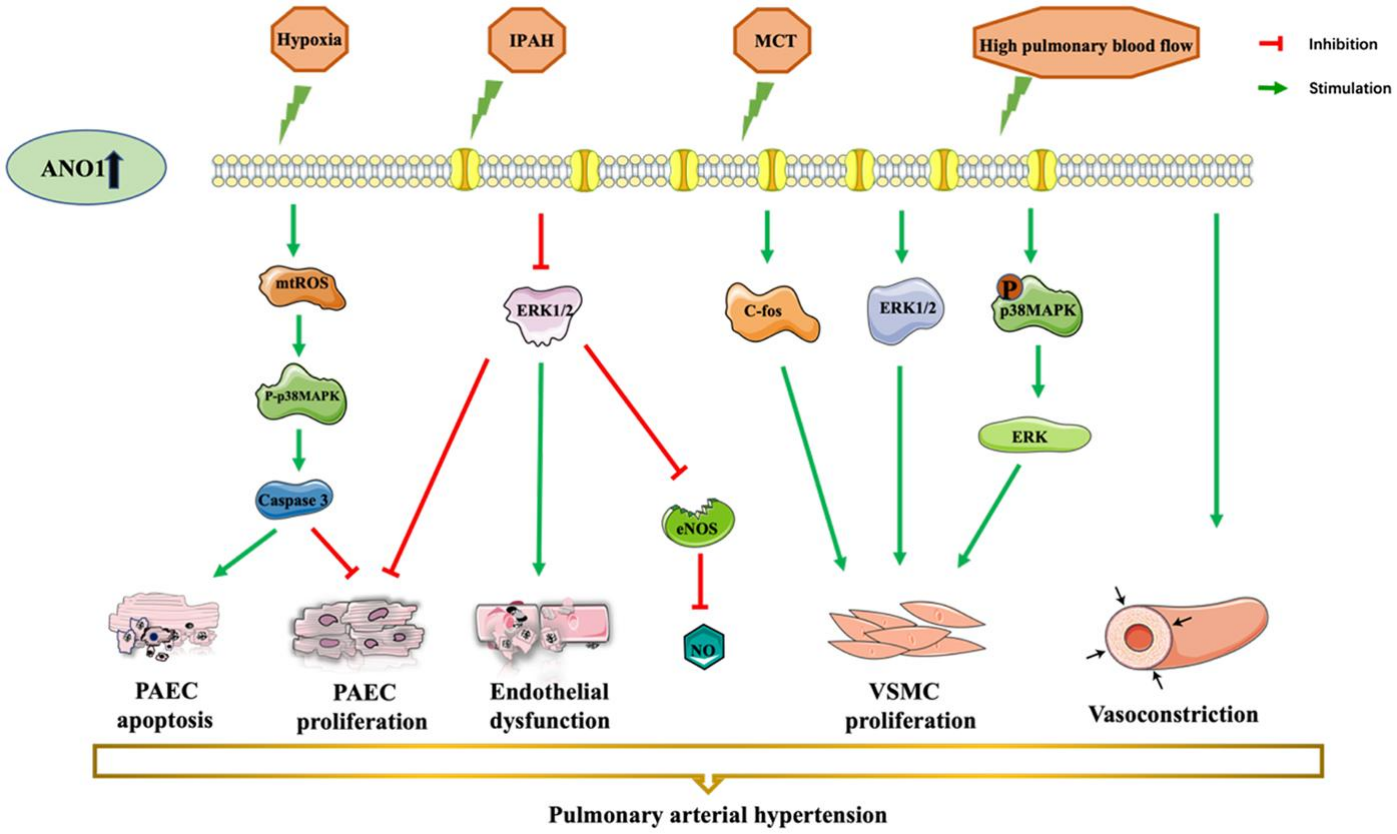
ANO1 dysfunction-induced vascular disorders and pulmonary arterial hypertension. ANO1 is upregulated in different models, in MCT- or chronic hypoxia-induced or high-flow-induced rat PAH model and human idiopathic PAH. There is a consensus that overexpression of ANO1 leads to endothelial dysfunction, PAEC apoptosis, PAEC proliferation, VSMC proliferation, and VSMC constriction. ANO1, Anoctamin-1; MCT, monocrotaline; NO, nitric oxide; PAEC, pulmonary artery endothelial cell; PASMCs, pulmonary artery smooth muscle cells; VSMC, vascular smooth cell.

Numerous studies have shown that ANO1 is involved in several pathological processes in the cardiocerebral vascular system: myocardial ischaemia/reperfusion injury (MIRI), arrhythmia, myocardial fibrosis, hypertension and stroke (as shown in Table 1). This review will present a detailed elucidation of the role of ANO1 in cardiocerebral vascular disease (as shown in Figures 2–4), providing a theoretical basis for the therapeutic targeting of ANO1 in treating these diseases (as shown in Table 2).

**Table 1:** Different roles of ANO1 in the cardiovascular disease.

Role	Model	Abnormal ANO1 expression	Results and mechanism	Reference
MIRI	Mouse I/R injury model and H9c2 H/R model	Upregulated	Upregulation of ANO1 induced MIRI via the miR-144-3p/ANO1/NLRP3 inflammasome axis	(26)
Arrhythmias	myocardial ischemia mice	Upregulated	Upregulation of ANO1 induced cardiac arrhythmia by accelerating phase 1 repolarization of action potentials and influencing cardiac action potential duration	(17)
Cardiac fibrosis	Rat cardiac tissues after MI and cardiac fibroblasts after hypoxia	Upregulated	Upregulation of ANO1 alleviated cardiac fibrosis by inhibiting TGF-β/smad3 pathway	(20)
	Pressure-overload mice (a model of thoracic aortic constriction) and ANO1 transgenic mice	Downregulated	Downregulation of ANO1 induced cardiac fibrosis, but overexpression of ANO1 alleviated it by inhibiting TGF-β/smad3 pathway	(21)
	Rat MI model	First upregulated and then decreased	Upregulation of ANO1 induced cardiac fibrosis via the AT1R-mediated MAPK signaling pathway	(31)
Systemic hypertension	SHR	Upregulated	Upregulation of ANO1 promoted VSMCs in arteries growth and proliferation and blood vessel constriction and induced hypertension	(50)
	AngII-infused hypertensive mice	Downregulated	Downregulation of ANO1 induced human aortic SMC proliferation and vascular remodeling by disrupting AngII-mediated KLF5/myocardin/SRF pathway	(51)
	AngII-infused hypertensive mice and SMC-specific ANO1 transgenic mice	Downregulated	Downregulation of ANO1 induced mice aortic SMC autophagy and exacerbated vascular remodeling by disrupting the formation of p62/Bcl-2/Beclin-1/VPS34 complex	(52)
	ANO1 endothelial-specific transgenic and knockout mice with AngII-infused hypertension	Not clear	Upregulation of ANO1 elevated BP and induced endothelial dysfunction by increasing Nox2-containing NADPH oxidase-derived reactive oxygen species, whereas the knockout of ANO1 produced opposite effects	(38)
	Pressure overload-induced myocardium remodelling of heart of mice	Upregulated	Upregulation of ANO1 promoted migration and angiogenesis of HUVECs	(53)
	HAECs	Downregulated	Downregulation of ANO1 promoted HAEC proliferation, migration, and angiogenesis	(55)
Pulmonary arterial hypertension	MCT-induced PH rats	Upregulated	Upregulation of ANO1 induced an elevation in Ca^2+^-activated Cl^−^ current	(57)
	IPAH and two animal models of PAH (the chronic hypoxic-induced mice and monocrotaline treated rats)	Upregulated	Upregulation of ANO1 stimulated vasoconstriction and proliferation of PASMC via enhanced c-fos phosphorylation	(36)
	MCT-treated PAH rats	Upregulated	Upregulation of ANO1 promoted PASMC proliferation, remodeling of pulmonary arterioles, and right ventricular hypertrophy by activating ERK1/2	(60)
	High-flow-induced pulmonary hypertensive rats	Upregulated	Upregulation of ANO1 promoted the proliferation of PASMCs via p38MAPK/ERK signal pathway	(59)
	PAECs isolated from IPAH	Upregulated	Upregulation of ANO1 promoted human PAEC apoptosis but suppressed PAEC proliferation via mtROS-p38-caspase-3 pathway	(45)
	PAECs isolated from IPAH	Upregulated	Upregulation of ANO1 inhibited human PAEC proliferation but unaffected HPAEC apoptosis and caused endothelial dysfunction via ERK1/2 pathway	(46)
Portal hypertension	BDL rat with PHT and PVSMC isolation and transfection	Downregulated in the portal vein of PHT rats *in vivo*	p-ERK1/2 was increased *in vivo* and ANO1 promoted PVSMC proliferation *in vitro*	(62)
	BDL mice with cirrhotic PHT and PPVL mice with non-cirrhotic PHT	downregulated in BDL-PVSMCs whereas not in PPVL-PVSMCs	Downregulation of ANO1 attenuated spontaneous contraction of the BDL-portal vein and alleviated PHT	(61)
Stroke	2k2c renohypertensive rats	Downregulated	ANO1 involved in AngII-induced cerebral vasoconstriction via the RhoA/ROCK signalling pathway	(65)
	Middle cerebral artery occlusion mice; OGD/R model	Upregulated	ANO1 expression was elevated in BCECs via lncRNA ENST00000530525 though recruiting transcription factors or enhancers after ischaemic stroke	(67)
	Middle cerebral artery occlusion mice	Upregulated	Upregulated ANO1 exacerbated brain injury via accumulating intracellular adhesion molecule-1 in a nuclear factor-kappaB-dependent manner	(66)
	BCECs isolated from mice	Not clear	ANO1 dysfunction stimulated BCEC proliferation and transendothelial permeability	(54)
	Mice stroke model	Not clear	Ischaemia-activated ANO1 increases pericyte contraction and reduces cerebral blood flow	(70)
	2-kidney,2-clip hypertensive rats	Downregulated	Downregulation of ANO1 induced BASMC proliferation and cerebrovascular remodeling by promoting cell cycle transition at the G0/G1 phase via the overexpression of cyclin D1 and cyclin E	(71)
	AngII-infused hypertensive mice	Downregulated	Downregulation of ANO1 by CaMKII*γ*-mediated phosphorylation induced BASMC proliferation and cerebrovascular remodeling	(72)
	Cultured rat BASMCs and VSMC-specific ANO1 transgenic mice	Not clear	ANO1 dysfunction promoted H_2_O_2_-induced BASMC apoptosis by increasing mitochondrial membrane permeabilization via interaction with CypD	(74)
	VSMC-specific ANO1 transgenic mice with AngII-induced hypertension	Not clear	ANO1 dysfunction inhibited AngII-induced BASMC migration and protected the basilar artery against remodeling by suppressing WNK1, RhoA/ROCK2/MLCP/MLC20, and integrinb3/FAK pathways	(75)
	VSMC-specific ANO1 transgenic mice with AngII-induced hypertension	Not clear	ANO1 dysfunction inhibited extracellular matrix deposition and AngII-induced cerebrovascular remodeling by reducing levels of MMPs/TIMPs and the activity of WNK1, TGF-β1/Smad3, ERK, and JNK	(76)

BASMC, basilar artery smooth muscle cell; BCEC, brain capillary endothelial cell; BDL, bile duct ligation; CVEC, cardiac vascular endothelial cell;H_2_O_2_, hydrogen peroxide; H/R, hypoxia/reoxygenation; IPAH, patients with idiopathic PAH; I/R, ischemia/reperfusion; KLF5, Kruppel-like factor 5; MASMC, mouse aortic smooth muscle cell; MCT, monocrotaline; MIRI, myocardial ischemia/reperfusion injury; PAEC, pulmonary arterial endothelial cell; PASMC, pulmonary arterial smooth muscle cell; PHT, portal hypertension; PPVL, partial portal vein ligation; PVSMC, portal vein smooth muscle cell; SRF, serum response factor; SHR, spontaneously hypertensive rat; VSMC, vascular smooth muscle cell.

**Table 2:** Profiling inhibitors of ANO1 and their potential pharmacological effects.

Name (alias)	Structure	IC_50_ (μmol/L)	Mechanism	Pharmacological effect	Special effect
T16A_inh_-A01	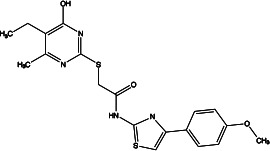	1.8	Inhibits Cl^−^ influx into nuclear and inhibits the activation of ERK1/2	Relaxes VSMC constriction (16, 50, 65); reduces BP (50); improves vascular remodeling and right ventricular hypertrophy (60); anti-cardiac fibrosis (22)	Concentration- and dose-dependent
CaCC_inh_-A01	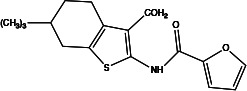	2.1	Inhibits ANO1 expression and causes a global decrease of Cl^−^ content; binding with ANO1 causes the collapse of the ion conduction pore (84);indirectly alters intracellular Ca^2+^ signalling (92)	Relaxes VMC constriction (83); anti-cardiac fibrosis (22)	Concentration- and dose-dependent; good pharmacokinetic characteristics; poor selectivity among ANO1 family proteins
MONNA	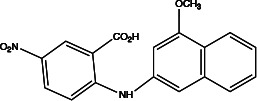	0.08	Inhibits Ca^2+^-activated ANO1 chloride currents; indirectly alters intracellular Ca^2+^ signalling (92)	Relaxes VSMC constriction (16, 83)	Concentration-and dose-dependent; in the presence and absence of Cl^−^
TM_inh_-23	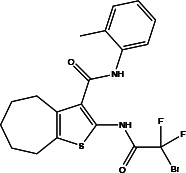	0.03	Inhibits Ca^2+^-activated ANO1 chloride currents *ex vivo* and *in vivo*	Reduces VSMC vasoconstriction and lowers BP (37)	Good selectivity and reversibility
Ani9	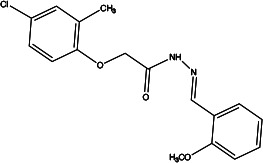	0.077	Inhibits ANO1 Cl^−^ current (85)	Attenuates VSMC contraction (16)	Dose-dependent; highly potency and selectivity; does not affect the intracellular Ca^2+^ signaling and CFTR Cl^−^ channel activity and ANO2
Tannic acid	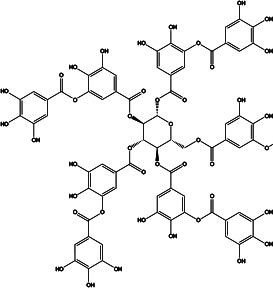	6	Inhibits Ca^2+^-activated ANO1 chloride currents	Relaxes aortic smooth muscle contraction (79)	Concentration- and dose-dependent; inhibits CaCCs in various cells but does not affect CFTR Cl^−^ channels and ENaC Na^+^ channels
Benzbromarone	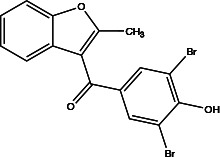	not clear	Inhibits Ca^2+^-activated ANO1 chloride currents *ex vivo* and *in vivo*	Vasodilates; reduces right ventricular pressure; attenuates remodeling of established PAH (36)	Dose-dependent; non-specific inhibitor
Nimodipine	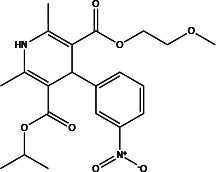	5	Decreases intracellular Ca^2+^ concentration and partly directly inhibits ANO1	Relaxes aortic smooth muscle (82)	Concentration-dependent
Niclosamide	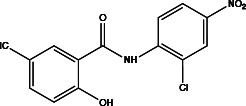	not clear	At positive membrane potentials and under conditions of elevated intracellular Ca^2+^ >2 μM inhibits ANO1	Relaxes VSMC constriction (86)	Concentration- and dose-dependent, strongly dependent on the membrane potential and the intracellular calcium concentration,inhibits CaV1.2

BP, blood pressure; IC_50_, the 50% inhibitory concentration; PAH, pulmonary arterial hypertension; VSMC, vascular smooth muscle cell.

## 2 The role of ANO1 in heart diseases

ANO1 is endogenously present in cardiac vascular endothelial cells (15), coronary VSMCs (16), ventricular myocytes (17, 18), atrial fibroblasts (19), and cardiac fibroblasts (20). Overexpression of ANO1 enhances depolarisation, increases the potential for Ca^2+^ channel opening, vasoconstriction, and ultimately decreases coronary perfusion, which can lead to ischaemic heart disease (16). Evidence suggests that ANO1 dysfunction is an emerging pathogenesis of MIRI, cardiac arrhythmias, and cardiac fibrosis (17, 20–22).

MIRI refers to increased damage to the ischaemic myocardium after restoration of blood flow (23, 24). The pathogenesis of MIRI is currently attributed to oxidative stress, Ca^2+^ overload, mitochondrial damage, and neutrophil activation (25). Initially, it was investigated that ANO1 was upregulated and miR-144-3p was downregulated, and then the NLRP3 inflammasome was activated during MIRI (26). Further study found that the upregulation of miR-144-3p inversely regulated ANO1 via binding to the 3′-untranslated region of ANO1 and suppressed the activation of NLRP3 inflammasome, which in turn attenuated NLRP3-mediated myocardial apoptosis and MIRI *in vivo* and *in vitro* (26). Therefore, the miR-144-3p/ANO1 axis may be a promising therapeutic target in myocardial ischaemia.

In addition, arrhythmia is a common manifestation with abnormal periodicity and regularity of myocardial electromechanical activity, particularly disturbed by various ion channels in the myocardial cell membranes (27). The increased expression and activity of ANO1 in mouse ventricular myocytes under conditions of myocardial ischaemia accelerate phase 1 repolarisation of action potentials and influence cardiac action potential duration, which partially leads to arrhythmias (17). Furthermore, the activation of ANO1 in cardiac fibroblasts affects the electrophysiological activity of cardiomyocytes via gap junctions, ultimately leading to ectopic pacing, which may be an underlying mechanism related to arrhythmia.

Cardiac fibrosis is a complex pathological process of increased myocardial stiffness and decreased systolic ejection (28). Cardiac fibroblasts (CFs), the most abundant cardiac cells, together with cardiomyocytes and vascular smooth muscle cells (VSMCs), make up the myocardium (29). In the progression and development of cardiac fibrosis, cardiac fibroblasts proliferate and differentiate into myofibroblasts under the influence of pathological factors that have enhanced their ability to secrete matrix metalloproteinases, proliferate, and migrate (30). Studies have shown that ANO1 expression is significantly increased during myocardial fibrosis. Gao et al. found that ANO1 expression in rat cardiac tissues increased significantly under hypoxic conditions after myocardial infarction (MI), especially in the border zone of the infarct and in cardiac fibroblasts (CFs) (20). Tian et al. further demonstrated that ANO1 expression peaked at weeks 1–2 after MI and then gradually declined, with these dynamic changes being closely related to the fibrosis process (31). Furthermore, *in vitro* experiments revealed that ANO1 expression increased in CFs under hypoxic conditions or TGF-β stimulation, indicating a potential role for ANO1 in the activation and differentiation of CFs (20, 32). However, the role of ANO1 in myocardial fibrosis is controversial. Tian et al. reported that ANO1 exacerbates cardiac fibrosis via the AT1R-mediated MAPK signalling pathway (31). ANO1 also affects the function of CFs by regulating intracellular Cl^−^ concentration and inhibition of ANO1 by both T16Ainh-A01 and CaCCinh-A01 could reduce the proliferation, migration, and collagen secretion of CFs in a dose-dependent manner by reducing Cl^−^ (22). Lately Tian et al. found that kockdown of ANO1 can significantly reduce the migration and adhesion ability of CFs, which may be due to the downregulation of integrin expression and reduced activation of focal adhesion kinase (32). But this study only used *in vitro* models of CFs and did not include *in vivo* experiments (e.g., animal models of myocardial infarction) to validate the pathological role of ANO1. Nevertheless, Gao et al. and Kong et al. revealed that the overexpression of ANO1 impeded the proliferation and differentiation of CFs, possibly via the inhibition of the TGF-β/smad 3 signalling pathway, which ultimately alleviated cardiac fibrosis *in vivo* MI modelling (20, 21). The focus of both papers is on myocardial tissue as a whole (comprising cardiomyocytes and fibroblasts, among others) through the construction of myocardial fibrosis model.

All in all, such disparities may be related to the diversity of experimental models, intervention times or microenvironments. We suppose that ANO1 may regulate fibrosis via distinct mechanisms at various pathological stages: initially, it mitigates fibrosis by curbing inflammatory responses; subsequently, it exacerbates fibrosis by stimulating the activation of CFs. This implies that precise modulation of ANO1 as a therapeutic target is imperative in specific pathological scenarios. Further studies are needed to investigate the molecular mechanisms of ANO1 in different cell types, as well as its potential clinical applications.

## 3 The role of ANO1 in hypertension

Hypertension is characterised by elevated blood pressure (BP) and vascular remodelling that can further damage target organs (heart, kidneys, brain, eyes, vasculature, etc.) and is influenced by environmental and genetic factors. Genetic diversity in ANO1 has been statistically associated with hypertension in humans, especially in males (33), although this has not been demonstrated by genome-wide sequencing studies (34). Accumulating evidence has proven that ANO1 plays an important role in the pathophysiology of hypertension, not only in vascular contraction but also in vascular remodelling, including changes in endothelial and VSMC function (35–38).

The mechanisms underlying the involvement of ANO1 in the initiation and progression of different types of hypertension are intricate and controversial. Substantial Cl^−^ accumulated in VSMCs due to the increased activity of Na^+^-K^+^-2Cl^−^ cotransporter 1(NKCC1) in hypertension and elevated the driving force for Cl^−^ efflux (as shown in Figure 2) (39). The cooperation between ANO1 and NKCC1 increased Cl^−^ efflux, potentiated membrane depolarisation and enhanced the open probability of Ca^2+^ channels, ultimately leading to an increase in BP (40). As for the sources of Ca^2+^, there are no clear and precise conclusions. Ca^2+^ from outside the SMCs entered the cell via voltage-gated L-type calcium channels or transient receptor potential canonical 6 channel (TRCP6), and some Ca^2+^ was released from the SR via IP_3_ and ryanodine receptors (41). In addition, ANO1 partially regulated arterial contractility by positively modulating the expression of the CACNA1C subunit of the L-type Ca^2+^ channels in tail arteries from transgenic mice (42). Moreover, TRPC6 and ANO1 channels were spatially localised in close proximity on the plasma membrane of cerebral artery myocytes, and TRPC6 channel activated a local intracellular Ca^2+^ signal that stimulated ANO1, thereby enhancing vasoconstriction and decreasing blood flow (43). Interestingly, ANO1 knockdown reduced the aorta contraction and potentially induced hypotension but increased tail and saphenous artery contraction by switching smooth cells to a pro-contractile phenotype, indicating that ANO1 is multifunctional for different parts of the vascular walls (44). Moverover, excess expression of ANO1 impaired endothelial function, possibly via the generation of reactive oxygen species (38, 45) and the reduction of nitric oxide (46), triggering vascular remodelling and ensued numerous cardiovascular diseases (different types of hypertension, stroke, etc.). Next, we elaborate on the role of ANO1 in different types of hypertension, including systemic hypertension, pulmonary arterial hypertension, and portal hypertension.

### 3.1 The role of ANO1 in systemic hypertension

The regulation of systemic arterial BP can be divided into long-term and short-term mechanisms. Long-term control is predominantly mediated by the kidneys through the regulation of sodium excretion and extracellular fluid volume (47). Short-term regulation is mediated by various cardiovascular reflexes that alter myocardial contractiliry and peripheral vascular resistance (48), and local autoregulatory mechanisms, such as the pressure-induced myogenic constriction of resistance vessels, which ensures stable blood flow to critical organs like the kidneys. Yip et al. discovered that ANO1 is expressed in afferent arterioles and is involved in pressure-induced myogenic contraction, indicating a role for ANO1 in short-term renal autoregulation (49). It is important to note, however, that the myogenic response primarily serves to buffer acute pressure fluctuations and has a limited role in setting long-term BP. This is supported by the finding that ANO1 knockout mice remain susceptible to salt-induced hypertension (35), underscoring that ANO1 is not part of the primary, kidney-dependent long-term control system. Beyond the kidney, ANO1 also influences blood pressure through systemic vascular tone. It is expressed in various vessels, including the resistance arteries, aorta, and carotid and mesenteric arteries of SHRs (50). Consistent with this distribution, conditional knockout of ANO1 or applying ANO1 inhibitor TMinh-23 reduced the responsiveness to vasoconstrictor stimuli, resulting in a reduction in systemic BP (35). The specificity of this mechanism is highlighted by the observation that TMinh-23 preferentially suppressed constriction in mesenteric resistance arteries compared to the aorta, conclusively demonstrating that the peripheral vasculature is the primary site of action for this blood pressure-lowering effect (37). It's interesting to note that the function and expression of ANO1 in the aorta has been altered in different animal models (50–52). ANO1 expression and activity are upregulated in aortic SMCs from spontaneously hypertensive rat (SHR) (37, 50), but downregulated in human aortic SMCs and mouse aortic SMCs from the AngII-induced hypertensive models (51, 52). These distinctions highlight the intricate pathophysiology of hypertension. In the SHR model, hypertension develops as a genetically determined primary condition that progresses gradually through vascular remodeling. Here, increased ANO1 expression may represent a functional and structural adaptation of VSMCs to sustained hypertensive stimuli, establishing a positive feedback mechanism that perpetuates elevated blood pressure (37). By contrast, hypertension in the acute AngII infusion model is primarily driven by the potent vasoconstrictor AngII, and the observed downregulation of ANO1 may act as a negative feedback response to mitigate excessive vasoconstriction and calcium overload. Thus, ANO1 expression appears to be highly context-dependent, shaped by the predominant etiological factors and the stage of hypertension development. The schematic mechanism of ANO1 function in vascular remodelling is shown in Figure 3. AngII markedly upregulated ANO1 expression in VSMCs of SHR in a concentration- and time-dependent manner through the AT1R/PI3 K/Akt pathway, which made VSMCs more sensitive to other agonists and exacerbated peripheral resistance and stimulated the growth and proliferation of VSMCs (50). However, in an AngII-induced hypertension model, AngII promoted Kruppel-like factor 5 (KLF5) expression and KLF5-myocardin binding, disrupted the feedback loop between ANO1 and myocardin, and then antagonised ANO1 transcription in human aortic SMCs, subsequently inducing cellular proliferation and vascular remodelling (51). Similary, reduced ANO1 expression in mouse aortic SMCs of AngII-induced hypertensive mice activated cell autophagy and exacerbated vascular remodelling by promoting p62/Bcl-2/Beclin-1/VPS34 complex formation (52).

Endothelial dysfunction, including endothelial proliferation, apoptosis, migration, and angiogenesis, is involved in the pathogenic process of various vascular diseases, in which ANO1 plays a multifaceted role (38, 45, 46, 53–55). It was found that ANO1 induced human umbilical vein EC dysfunction by inhibiting Nox2 degradation and increasing Nox2-containing NADPH oxidase activity, resulting in increased reactive oxygen species generation during AngII-infused hypertension, whereas knockout endothelial-specific ANO1 reverse the hypertensive effect (38). Moreover, Zhang et al. also revealed that ANO1 positively induced the migration and angiogenesis of human umbilical ECs under normal or under stress conditions *in vitro* (53). In contrast, ANO1 inhibition by cholesterol and cholesterol-induced DNA methyltransferase 1-mediated methylation facilitated human aortic EC proliferation, migration, and angiogenesis, indicating that ANO1 was negatively correlated with human aortic EC activity (55). Thus, activation of ANO1 limited endothelial angiogenesis during high cholesterol, which was beneficial in diseases with high cholesterol pathological states such as atherosclerosis (55).

### 3.2 The role of ANO1 in pulmonary arterial hypertension

Pulmonary arterial hypertension (PAH) is a progressive multifactorial disease with vasoconstriction and hyperproliferation of pulmonary artery endothelial cells (PAECs) and pulmonary artery smooth muscle cells (PASMCs), ultimately leading to right heart failure (56). ANO1 was upregulated in the PASMCs from monocrotaline- or chronic hypoxia-induced or high-flow-induced rat PAH model and in patients with idiopathic PAH (36, 57–59), contributing to vascular constriction and increased blood pressure (as shown in Figure 4) (36, 57).

ANO1 was positively correlated with PASMC proliferation in PAH (36, 59, 60). Increased expression and activity of ANO1 stimulated human PASMC proliferation during idiopathic PAH by increasing c-fos phosphorylation, which can be suppressed by benzbromarone and blocking or silencing of ANO1 (36). Another research found that ANO1 facilitated PASMC proliferation, pulmonary arterioles remodelling and right ventricular hypertrophy, possibly via activating ERK1/2 in an MCT-treated hypertensive rat model (60). Moreover, further investigation showed that ANO1-induced proliferation of PASMCs may be mediated by the p38MAPK/ERK signalling pathway in high-flow-induced PAH rats (59).

Previous studies have shown that ANO1 was distributed in the mitochondria and plasma membranes in human PAECs (45, 46). The augmented expression and activity of ANO1 promoted mitochondria-dependent PAEC apoptosis via a mtROS-p38-caspase-3 pathway, suppressing PAEC proliferation in idiopathic PAH patients (45). However, Skofic Maurer et al. studied that enhanced ANO1 expression and activity contributed to endothelial dysfunction, decreased PAECs proliferation but did not affecte apoptosis, and reducted nitric oxide likely through activating the ERK1/2 pathway (46). The difference between the two mechanisms may be due to the cellar heterogeneity and experimental conditions. Further studies investigating the role of ANO1 in endothelial cells are warranted.

### 3.3 The role of ANO1 in portal hypertension

Portal hypertension is a group of syndromes caused by persistent elevation of portal pressure, the vast majority of which are caused by cirrhosis of the liver. ANO1 contributed to spontaneous contractions, a significant stimulus of blood flow from the mesenteric vascular bed to the liver (61). Recently, several investigators found that ANO1 was downregulated in portal smooth muscle cells (PVSMC) from bile duct ligation mice with cirrhotic portal hypertension via increased AngII, followed by a reduction in spontaneous contractions (61). ANO1 is also involved in PVSMC proliferation and hyperexpression of ANO1 facilitates PVSMC proliferation *in vitro* but inhibits PVSMC proliferation from portal hypertensive rats *in vivo*, suggesting that ANO1 may also be a negative modulator of PVSMC proliferation under the influence of multiple *in vivo* factors (62). More research is necessary to establish whether ANO1 is a viable candidate for the treatment of portal hypertension.

## 4 The role of ANO1 in stroke

Stroke, a cerebrovascular accident, comprises a group of disorders resulting from a sudden blocked or ruptured blood vessel in the brain, causing damage to brain tissue. It is widely recognised that vascular remodelling of cerebral arterioles caused by hypertension is a major factor contributing to the increased incidence of ischemic stroke due to the narrowing atrial and reduced blood flow (63). ANO1 participates in the regulation of myogenic tone via non-selective cation channel-generated local Ca^2+^ signalling in cerebral arteries when membrane distension, including cell swelling and pressure-induced membrane stretch (64). Activated ANO1 enhanced AngII-induced phosphorylation of MYPT1 and MLC through the RhoA/ROCK signalling pathway, which triggered basilar vasoconstriction in male Sprague-Dawley rats (65). To improve our understanding of new approaches to control stroke development and ameliorate blood-brain barrier (BBB) damage, it's necessary to elucidate the pathological role of ANO1 in cerebrovascular remodelling during the development of hypertension and BBB injury after stroke (as shown in Figure 3).

Researchers found that ANO1 is expressed in brain capillary endothelial cells (BCECs) and is upregulated after ischaemic stroke caused by middle cerebral artery occlusion or under hypoxia (54, 66). A recent study demonstrated that the lncRNA ENST00000530525 may affect ANO1 expression by recruiting transcription factors or enhancers after ischaemic stroke (67). Inhibiting or silencing ANO1 alimerate ischemic-induced BBB injury via downregulating intracellular adhesion molecule-1 and neutrophil accumulation in a nuclear factor-kappaB-dependent manner, indicating that downregulation of ANO1 protects BBB disruption after ischemia stroke (66). In addition, the function of ANO1 regulates the proliferation and migration of BCECs, thus ensuring the maintenance of the barrier function of the BBB (68). This team further studied that the upregulated expression of ANO1 stimulated the proliferation and transendothelial permeability of BCECs, which may lead to BBB dysfunction under hypoxic stress (54). The administration of the ANO1 inhibitor T16Ainh-A01 or siRNA attenuated these effects. Contractile pericytes, a vital component of the BBB, serve as principal regulators of capillary diameter and cerebral blood flow (69). Following ischemic events, these pericytes augment capillary constriction and diminish cerebral blood flow through ANO1-mediated Ca^2+^-activated chloride efflux. Targeting ANO1 with Ani9 or deletion promotes pericyte relaxation and slows pericyte loss, attenuating the microvascular blood flow and BBB function (70).

Cerebrovascular remodelling is accompanied by increased proliferation and migration of VSMCs, deposition of ECM, and inhibition of VSMC apoptosis. Numerous studies concluded that ANO1 was negatively associated with cerebrovascular remodelling during hypertension. ANO1 expression and activity are decreased in basilar artery smooth muscle cells (BASMCs) from 2-kidney, 2-clip(2k2c) renohypertensive rats (71) and AngII-infused hypertensive mice (72), which are negatively regulated by the enhanced CaMKII*γ* activity via phosphorylation of serine 727 within ANO1 in basilar arteries. One study revealed that AngII suppressed the expression of ANO1 in BASMCs from 2k2c renohypertensive rats and subsequently accelerated vascular remodelling, with the underlying mechanism being that AngII increased endophilin A2 and subsequently enhanced ubiquitin-mediated degradation of the ANO1 protein (73). The reduction of ANO1 promotes BASMC proliferation and cerebral vascular remodelling by promoting the G1/S transition via increasing cyclin D1 and cyclin E expression without affecting the cell cycle negative regulators p21 and p27 (71, 72).

In addition to inhibiting abnormal BASMC proliferation, VSMC-specific transgenic expression of ANO1 also exerts a protective effect on BASMC apoptosis, migration, and extracellular matrix accumulation (74–76). Zeng et al. investigated that ANO1 contributed to hydrogen peroxide-induced mitochondria-dependent BASMC apoptosis from rats and mice via interaction with CypD, which could be alleviated by silencing ANO1 and applying T16A_inh_-A01 (74). In addition, ANO1 suppressed WNK1, which in turn inhibited the RhoA/ROCK2/MLCP/MLC20 and integrinb3/FAK pathways, thereby antagonising AngII-induced BASMC migration and protecting the basilar artery from remodelling during hypertension (75). Further investigation showed that the ANO1 decreased the activation of WNK1 and subsequently reduced the levels of MMPs, which was followed by attenuation of the activity of the typical TGF-β1/Smad3, non-typical TGF-β1/ERK and JNK pathways, ultimately protecting against extracellular matrix deposition and antagonising AngII-induced cerebrovascular remodelling in mice *in vivo* and *in vitro* (76). It may be a potential target for treating ischemic stroke and ischemia-induced BBB damage, but this effect should be tested in the whole model, not just the cerebrovascular one. It would be interesting to see further studies aimed at validating ANO1 as a therapeutic target for brain stroke.

## 5 ANO1-targeted pharmacological modulation and therapy

Following the diverse functions of ANO1 mentioned above, applying ANO1 modulators should be cautious and specific in different cardiocerebral vascular diseases. A growing body of literature has investigated the application of ANO1 inhibitors in the cardiocerebral vascular system, especially anti-hypertension or anti-cardiac diseases (77). The inhibitors are divided into two categories: natural chemicals and synthetic agents.

Several compounds from natural daily foods have been shown to protect against cardiovascular disease, including vegetables, fruits, cereals, green tea, and red wine (78, 79). Plant lignan compounds, such as kobusin and eudesmin, show an antihypertensive effect in patients in clinical trials (80), which partially explains the therapeutic effect of ANO1 active inhibitors *in vitro* and *in vivo* (78). In addition, green tea and red wine had cardioprotective effects, underlying that tannic acid, related gallotannins, and polyphenols are potent and effective ANO1 inhibitors (79). However, the 50% inhibitory concentration (IC_50_) of these natural inhibitors was higher than that of chemical inhibitors, possibly due to the lack of purity of the ANO1 inhibitors.

The discovery of new therapeutic avenues from drugs already on the market is a viable strategy for obtaining safe and effective therapeutic drugs (81). Several clinical agents have been discovered that exhibited inhibitory effects on ANO1, particularly benzbromarone and nimodipine (as shown in Table 2) (36, 82). Benzbromarone, a gout drug, lowered right ventricular pressure, reduced elevated PAH, and attenuated vascular remodelling in established PAH in a dose-dependent manner. The application of benzbromarone in treating hypertension, especially PAH, is considered a priority in the clinic (36). Nimodipine, an LTCC blocker, inhibited ANO1 indirectly by decreasing intracellular Ca^2+^ concentration or directly in a dose-dependent manner, and interestingly, the effect on relaxation of aortic VSMC contraction was reversible since the inhibitory effect disappeared completely 8 min after removal of nimodipine (82).

Previous studies have shown that there is a new class of ANO1 antihypertensive blockers, such as T16A_inh_-A01 (50), CaCC_inh_-A01 (83, 84), MONNA (83), TM_inh_-23 (37), Ani9 (85), Niclosamide (86) (as shown in Table 2). T16A_inh_-A01 could reduce VSMC constriction and local BP elevation in SHRs (50) and alleviate Ang II-induced rat basilar arteries constriction (65). Additionally, T16A_inh_-A01 could reverse PAH and ameliorate small pulmonary artery remodelling and right ventricular hypertrophy, possibly inhibiting ERK phosphorylation (60). It was demonstrated that TM_inh_-23 was one of the most potent inhibitors of ANO1 according to the minimum IC_50,_ which was approximately close to 30 nM (87). It reduced vasoconstriction *in vitro* and lowered systolic and diastolic BP in SHR *in vivo* without a rebound effect after withdrawal but produced a transient increase in heart rate (37). Furthermore, MONNA, T16A_inh_-A01, and Ani9 also impaired coronary artery constriction and increased coronary flow and may be potential development candidates for drug therapy for ischaemic heart disease and MI (16). Among them, T16A_inh_-A01 and CaCC_inh_-A01 could suppress CFs proliferation, migration, and collagen secretion in a dose-dependent manner and may serve as novel anti-cardiac fibrotic therapeutics in the future (22).

Recent advances in potent and selective chemical probes and drug candidates targeting specific ANO1 will enable study designs that carefully incorporate targeted genetic approaches, including human induced pluripotent stem cells, condition/site-specific knockout in mice, or CRISPR gene editing in native cells (88). A new strategy may be to treat ANO1-related diseases by targeting small biological molecules. For example, the miR-144-3p/ANO1 axis may be a promising therapeutic target in myocardial ischaemia (26, 89). In addition, endophilin A2 and ET-1 have provided novel mechanistic insights and therapeutic targets for ANO1-induced vascular remodelling and BP elevation (73, 90). Moreover, alternative splicing of the primary ANO1 transcript could generate multiple variants that were altered in voltage-dependent and Ca^2+^ sensitivity and the rate of channel activation and deactivation, suggesting a putative therapeutic regimen (15).

Nevertheless, the pharmacological regulation and potential therapeutic strategies related to ANO1 remain limited. Firstly, ANO1 is present in different tissues and organs, and the structure of ANO1 is similar to other ion channels (ANO2, GFTR, ENaC, BEST1, etc.) (91), suggesting that the drugs acting on ANO1 may have off-target or non-specific effects. Secondly, although several ANO1 inhibitors have been discovered by high-throughput chemical library screening, the specific binding sites and interaction mechanisms are not addressed. The search for new potent and highly selective ANO1 inhibitors is still in its infancy, and most ANO1 inhibitors have only been tested in cell or animal experiments. Future studies are required to obtain insight into the precise mechanism of these molecules acting on ANO1 and to explore more organ- and tissue-specific compounds, which especially need to be validated in clinical trials.

## 6 Challenges for clinical use

There is still a long way to go to elucidate the mechanisms and clinically turn ANO1 into therapeutic targets for cardiocerebral vascular dieases. Firstly, there is a discrepancy in the expression of ANO1 in different models with different humoral factors. For all laboratory forms and models of hypertension, a complete investigation of the effects of blocking ANO1 to reduce BP is needed, as this may have unfavourable outcomes in cerebral and portal microcirculation. Secondly, the role of ANO1 in several pathophysiological cardiocerebral vascular progresses remains complex and contradictory. Especially in cardiac fibrosis under different experimental conditions, overexpression of ANO1 inhibits cardiac fibrosis by inhibiting the TGF-1 pathway, while another team verified that ANO1 promotes cardiac fibrosis via the AT1R-mediated MAPK signalling pathway. How ANO1 interacts with the MAPK pathway and the exact upstream and downstream relationships remain are unclear. Therefore, there is an unmet need for further cell and animal experiments to determine the precise signalling pathways involved in ANO1-associated cardiac fibrosis. Finally, there is an urgent need to systematically elaborate the crystal structure of ANO1 and its modulators, which will require much-needed effort and technological innovation to understand the structure-function relationship, physiological action, and pharmacological modulation of ANO1. The novel ANO1-targeted therapeutic approaches will be widely applied to cardiocerebral vascular disease in the future.

## 7 Conclusions

The ANO protein family is emerging as a fascinating class of ion channels, particularly because of their Ca^2+^ dependence and voltage sensitivity. The widespread distribution and involvement of ANO1 in multiple interferences in cardiocerebral vascular systems, in combination with the improvement of significant adverse effects of ANO1 knockout or ANO1 channel inhibition, render ANO1 an attractive therapeutic target. Herein, we present an overview of ANO1 and ANO1-related cardiac and cerebrovascular disease, emphasise that the disturbance of ANO1 is involved in the pathogenesis of the cardiac and cerebrovascular disease, and summarise existing and potential treatments targeting ANO1 for cardiac and cerebrovascular disease. Hopefully, this review will lay the groundwork for a deeper understanding of the cardiac and cerebrovascular mechanisms and treatments associated with ANO1 in the future.
